# Patients Are Paying Too Much for Tuberculosis: A Direct Cost-Burden Evaluation in Burkina Faso

**DOI:** 10.1371/journal.pone.0056752

**Published:** 2013-02-25

**Authors:** Samia Laokri, Maxime Koiné Drabo, Olivier Weil, Benoît Kafando, Sary Mathurin Dembélé, Bruno Dujardin

**Affiliations:** 1 School of Public Health, Université Libre de Bruxelles, Research Center Health Policy and Systems - International Health, Brussels, Belgium; 2 Laboratoire National de Santé Publique (LNSP), Ouagadougou, Burkina Faso; 3 HLSP Institute, London, United Kingdom; 4 Direction Régionale de la Santé (DRS) du Plateau Central, Bureau du Suivi-Évaluation, Ouagadougou, Burkina Faso; 5 Programme National de Lutte Antituberculeuse (PNLAT), Ouagadougou, Burkina Faso; Institut de Pharmacologie et de Biologie Structurale, France

## Abstract

**Background:**

Paying for health care may exclude poor people. Burkina Faso adopted the DOTS strategy implementing “free care” for Tuberculosis (TB) diagnosis and treatment. This should increase universal health coverage and help to overcome social and economic barriers to health access.

**Methods:**

Straddling 2007 and 2008, in-depth interviews were conducted over a year among smear-positive pulmonary tuberculosis patients in six rural districts of Burkina Faso. Out-of-pocket expenses (direct costs) associated with TB were collected according to the different stages of their healthcare pathway.

**Results:**

Median direct cost associated with TB was US$101 (n = 229) (i.e. 2.8 months of household income). Respectively 72% of patients incurred direct costs during the pre-diagnosis stage (i.e. self-medication, travel, traditional healers' services), 95% during the diagnosis process (i.e. user fees, travel costs to various providers, extra sputum smears microscopy and chest radiology), 68% during the intensive treatment (i.e. medical and travel costs) and 50% during the continuation treatment (i.e. medical and travel costs). For the diagnosis stage, median direct costs already amounted to 35% of overall direct costs.

**Conclusions:**

The patient care pathway analysis in rural Burkina Faso showed substantial direct costs and healthcare system delay within a “free care” policy for TB diagnosis and treatment. Whether in terms of redefining the free TB package or rationalizing the care pathway, serious efforts must be undertaken to make “free” health care more affordable for the patients. Locally relevant for TB, this case-study in Burkina Faso has a real potential to document how health programs' weaknesses can be identified and solved.

## Introduction

Direct cost-burden of illness, and particularly for a chronic disease such as tuberculosis, can cause delays, slower recovery, exacerbate health problems and drug resistance. Moreover, it may lead to catastrophic health expenditures and impoverishment as a result of the use of health services [1]. Protecting people from this financial risk is definitely a priority concern of policy-makers [2]–[4]. This is why free-of-charge health programs have been implemented, such as the TB control strategy. And yet, the costs for patients of TB treatment have largely been ignored [4].

Despite positive global progress, the international Stop TB Partnership targets of reducing TB prevalence and mortality will not be met in Africa [2]. In most Sub-Saharan African countries, and for instance in Burkina Faso, poverty and weak health systems remain a fertile breeding ground for tuberculosis and are likely to remain so in the coming years. In particular, poor case detection and treatment are jeopardizing the impact of National Tuberculosis Control Programmes (NTPs) and generating new challenges such as the HIV/AIDS co-infection, and the growth of multidrug-resistant tuberculosis. These factors complicate treatment and undermine the efficacy of the program and the achievement of targeted objectives.

While potential financial barriers have been stated as the rationale for implementing a free-of-charge strategy, the population still faces lingering and underestimated out-of-pocket expenses [5]. Therefore access to TB care is still challenging. The purpose of this study was to estimate direct costs (out-of-pocket expenditures) of TB care and control from the patient perspective and evaluate whether they are prohibitive or not. We aimed at describing direct costs in order to feed a discussion on the possible ways to mitigate financial obstacles and enhance performances of the TB care and control strategy.

## Materials and Methods

### Ethics statement

The study was carried out according to the international and national standards and was approved by the National Ethics Committee: “Comité national d'éthique pour la recherche en santé (CNERS), Ministère de la Santé 03 BP 7009. Ouagadougou 03. Burkina Faso.” Informed consent was systematically requested. All subjects participating in the study signed a voluntary consent form after being given all the information necessary and sufficient to make an informed decision regarding their participation in this study.

### Study Setting

The present study was conducted in six rural health districts of central Burkina Faso (Bousse, Koupela, Ouargaye, Zabre, Ziniare and, Zorgho) covering a population of nearly 1,447,000 inhabitants (www.insd.bf). The national TB control strategy is based on the DOTS implemented nationwide through a network of public CDTs (*Centres de Diagnostic et de Traitement*) located at health district level. Beforehand, identification of TB suspects was performed by nurses during a consultation at the first-line health centers (FLHCs). Then, suspected tuberculosis patients were referred from first-line health centres to the CDTs where the diagnosis was confirmed and the treatment prescribed. Diagnosis was based on a series of 3 sputum smear microscopies and required at least 4 contacts before initiation of the treatment. The DOTS strategy consists in a two-month intensive treatment (during which the drugs are delivered in the CDT on a daily basis) followed by a four-month continuation treatment (during which the patient comes weekly to the CDT to get the drugs and for clinical control). In January 2008, the national program had changed its treatment regimen from an eight-month to a six-month regimen resulting in a two-month reduction of the continuation treatment. A direct supervision from health workers for daily drug administration is compulsory during intensive treatment. During continuation treatment, a stock of drugs (covering up to one month of treatment) is regularly given to each patient for home based treatment. Drug collection visits during continuation phase could take place either at the CDT or at the FLHC closest to home. Control examinations of sputum were undertaken during the treatment follow-up. A second line re-treatment is started in case the smears are positive. Under DOTS, TB diagnosis (based on sputum smear microscopy) and regimen are provided free-of-charge.

### Study design and participants

A cross-sectional study was performed systematically among smear-positive TB patients enrolled in the NTP between June 2007 and June 2008. Data collection was nested within the European funded FORESA project which supported operational research on tuberculosis and health systems related issues in West Africa between 2006 and 2009 [6], [7].

Questionnaires were available in both French and the local language and were pre-tested. After informed consent, in-depth interviews lasting on average 3 hours were held among 242 sputum smear-positive patients being treated or having completed their treatment in the last 6 months. The trained interviewers worked in pairs to guarantee quality of the data. They were supervised by a field coordinator under close guidance of the research team. By considering the whole TB care pathway, we covered the period from onset of symptoms to completion of the treatment. Data were collected and referred to the following stages: pre-diagnosis (from onset of symptoms to first visit to health facility), diagnosis (from first visit to health facility to diagnosis confirmation), treatment initiation (from diagnosis to beginning of treatment) and, finally intensive and continuation treatment (from start to end of treatment).

The cost items included in the present study related to seeking diagnosis and treatment and referred to medical and non-medical expenses (i.e. examination and laboratory tests, consultation fees, drugs, hospital care, transportation costs to reach health providers, services provided by traditional healers and food supplements). All kinds of costs (including payments in kind) were systematically explored through the successive stages of the patient care pathway. Out-of-pocket expenses (direct costs) were initially expressed in local currency (FCFA) and then converted in US dollars (US$) using OANDA Rates™: 655.957 CFA BCEAO Franc (XOF) = 1 Euro (EUR) = 1.459 US$ (Mean study period price).

### Data processing

The data capture was performed using EPI-2000 (Centre for Disease control and Prevention, Atlanta, GA, USA). Data management and statistical analysis were processed with the IC/STATA 10 for Windows statistical package. Summary statistics (mean and standard deviation or median and interquartile range for continuous data and frequency distributions for categorical variables) were used to describe the study sample and cost-burden factors.

## Results

### Patients' characteristics

Verification of the quality and consistency of data resulted in the selection of 229 patients (i.e. 95% of the 242 interviewed). The average age of the study population was 41.5 years. It included 153 males (66.8%) and 76 females (33.2%). The demographic and clinical characteristics of the patients are summarized in Table 1 while the socioeconomic factors are presented in Table 2.

**Table 1:** Patients' demographic and clinical characteristics (n = 229).

Category	Subcategory	Result (% (n))
Gender	Male	66.8 (153)
	Female	33.2 (76)
Age (missing = 1)	Age (Mean (SD))	41.5 (16,5)
Household size	<3	35.4 (81)
	[3]–[5]	13.5 (31)
	>5	51.1 (117)
TB treatment category	Treatment completed cases	52.8 (121)
	Under first line treatment cases	44.1 (101)
	Under second line treatment cases	3.1 (7)
TB/HIV status (missing = 44)	Coinfected	14.1 (26)

**Table 2:** Patients' socioeconomic characteristics (n = 229).

Category	Subcategory	Result (% (n))
Education	Not attended	64.7 (147)
	Primary School	14.8 (34)
	Being literate	14.8 (34)
	Secondary School	6.1 (14)
Occupation (missing = 26)	Farming or cattle farming	52.2 (106)
	Housework	24.1 (49)
	Small Business	18.2 (37)
	Without occupation	4.4 (9)
	Civil servant	1.0 (2)
Type of housing (missing = 3)	Mud brick houses	61.9 (140)
	Mud brick & cement houses	35.0 (79)
	Cement houses	3.1 (7)
Home water source (missing = 64)	Yes	11.5 (19)

Most of the patients (86%) benefitted from the support of close relatives (transportation and/or labour substitution). A majority of patients (58.5%) lived less than 5 kilometres from a FLHC and over a third (36.4%) lived less than 5 kilometres from a CDT.

### Overall direct costs

From onset of symptoms to end of treatment, overall median direct cost incurred by TB patients was US$101.1 (53.1–172.4) as a consequence of fees, drug costs during care-seeking paid outside public health facilities, medical costs induced by TB control practitioners, traditional healers' charges or transportation and food costs.

### Patient care pathway analysis

**Figure 1:**
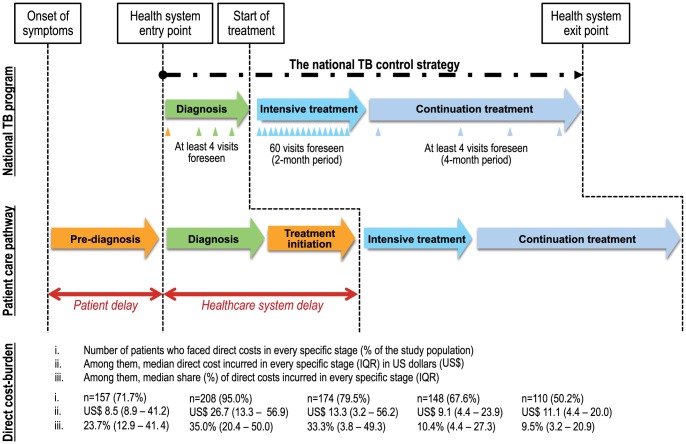
Direct cost-burden analysis for Tuberculosis. A patient care pathway analysis was used to estimate the extent, level and distribution of direct costs associated with TB (n = 229). By breaking down direct costs of the successive stages of the TB patient pathway in rural Burkina Faso, the figure highlights the gap between the national TB control strategy and the effective patient care pathway. Most patients accumulated medical and non-medical out-of-pocket expenses at every single stage of their care pathway. Moreover, it showed three kinds of delays which were respectively patient delay in the pre-diagnosis period, provider delay related to diagnosis, and treatment delay related to treatment initiation stage.

By breaking down direct costs of the successive stages of the TB patient pathway, Figure 1 highlights the gap between the national TB control strategy and the effective patient care pathway. Direct costs were assessed within this framework: most patients accumulated medical and non-medical out-of-pocket expenses at every single stage of their care pathway. We therefore presented the magnitude of direct costs for them. Moreover, it showed three kinds of delays which were respectively patient delay in the pre-diagnosis period, provider delay related to diagnosis, and treatment delay related to treatment initiation stage.

#### Pre-diagnosis (patient delay)

The median direct cost specific to the pre-diagnosis stage corresponded to US$8.5 (8.9–41.2) per patient (n = 157). Out of the 229 patients, patient delay ranged from less than a week to more than 3 months with a majority (57.2%) within 1 month. Before entering the healthcare system, the median number of health encounters in its fullest sense (including non-conventional sector) was 3 (mean = 4.3). Definitely, 71.7% of patients (n = 157) were already facing direct costs associated with TB (i.e. self-medication, transportation, traditional healers' services). Half of the latter spend at least 23.7% (12.9–41.4) of their total direct costs before the first visit to a FLHC.

#### Diagnosis (provider delay)

The median direct cost related to diagnosis stage was US$26.7 (13.3–56.9) per patient. For most of the patients (83.8%), provider delay lasted less than 2 weeks. Compared to the 4 visits required by the national strategy, 16.6% of patients consulted public providers even more. But most striking is that 95% of patients (n = 208) faced out-of-pocket expenditures during the diagnosis process (i.e. fees, travel costs paid to reach various providers, sputum smears microscopy and chest radiology). Among them, the median share of overall direct costs incurred for this stage reached 35.0% (20.4–50.0).

#### Treatment initiation (treatment delay)

For treatment initiation, the median direct cost was US$13.3 (3.8–49.3) per patient (n = 174). A majority (52.0%) of the 229 patients faced until 3-week delay before starting their treatment. In contrast with the national strategy, they were not put on treatment on the same day as diagnosis. For them, the number of additional travel to seek care ranged from 1 to 11. As a consequence, there are extensions and complications of the treatment leading to additional direct costs (mainly travel costs). Beyond this first observation, between diagnosis confirmation and start of the treatment, 79.5% of patients (n = 174) faced some expenses (i.e. travel costs, traditional healers) and half of them spent 33.3% (3.8%–49.3%) of their total direct costs.

#### Intensive treatment

During intensive treatment, the median direct cost was US$9.1 (4.4–23.9) per patient. Most patients (98.7%) followed the intensive treatment without interruption and few (1.3%) discontinued their intensive treatment for financial or family reasons. Even during intensive treatment where regimen is delivered free-of-charge, 67.9% of patients (152) faced some medical and/or non-medical costs. Among them, 98.0% (149/152) had to support non-medical costs such as food and transportation and 28.3% (43/152) had to finance medical costs such as hospitalization, user fees, chest X-rays, extra sputum tests (i.e. BAAR sputum test control) or complementary medical examinations. The median share of overall direct costs incurred for intensive treatment was 10.4% (4.4–27.3).

#### Continuation treatment

The median direct cost during continuation treatment was US$11.1 (4.4–20.0) per patient. Most patients (99.1%) followed the continuation treatment without interruption. As seen previously for intensive treatment, for continuation treatment, a majority of 50.2% (110) had to face spending during this stage. Among them, all patients (110/110) faced non-medical expenditures such as food and transportation expenses and 24.5% (27/110) faced medical expenditures such as drugs or small medical equipment. The median share of overall direct costs incurred for continuation treatment was 9.5% (3.2%–20.9%).

### Can poor patients really afford free TB care and control?

Out of the 229 patients, almost half (45.9%) of individual incomes were under the national poverty line (i.e. US$15.3 per adult per month) [8]. This proportion rose to 86.5% (n = 198) when taking into account the minimum salary as a threshold (i.e. US$68.3 per adult per month). Male sex bias was showed for the median individual income (p-value = .003). However, there was no sex bias for median direct costs (p-value = .48), nor for median cost-burden associated with TB (i.e. percentage of household's income) (p-value = .80).

For half of the patients, overall direct costs represented more than 2.8 months of their own household income (1.1–5.2). By extrapolation and based on a linear estimation of household income, this median represented 23% of annual household income. This might get worse for those whose incomes were highly volatiles, i.e. for 29.2% of households (65).

## Discussion

Our findings have shown that direct cost-burden of TB borne at household level in rural Burkina Faso is substantial and prohibitive. It suggests that the poorest would unlikely be able to finance TB without diverting basic resources. Overall out-of-pocket expenses associated with TB (i.e. Median direct costs of US$101 per patient) go well beyond what the national program currently covers. In the same period, in Nigeria, a similar research reached congruent findings (i.e. Median direct costs of US$94 for ambulatory TB patients) [9]. Indeed, exemptions for TB fees often only apply to the first three sputum tests and visits, drug regimen and clinical control. Nevertheless, other hidden costs still arise from the TB care pathway such as additional medical examinations, laboratory tests and X-rays or even travel costs. These are not supplied. Moreover, linked to poor financial resources of health services, overcharging practices may have occurred for services that were supposed to be free-of-charge [10]–[12] and can explain in part those extra-costs. Consequently, most of the patients who already live in extreme precariousness, are dangerously at risk of catastrophic TB expenditures, usually leading to deeper hazardous impoverishment of the patient's household [13], [14]. Whereas TB may occur for a limited period in lifetime, even if it is chronic, socio-economic consequences resulting from its case management tend to be permanent and impede poverty [14].

Our demonstration that the poor spend almost one fifth of their total direct expenses before diagnosis is in line with the assumption of Kemp et al suggesting that relative economic burden among poor was higher than for the richer [15]. Often neglected, yet it appeared important to draw attention to care-seeking behaviours during pre-diagnosis period [16] and diagnosis period [9], especially as a great part of expenses arose because of delayed referral for TB diagnosis. The uppermost burden related to median share of overall direct costs occurred before starting the treatment (i.e. up to 35% for diagnosis only). Supporting this observation, evidence from Nigeria reported much higher direct costs before and during diagnosis (i.e. respectively US$29 and US$31 versus US$17 for post-diagnosis period) [9]. Likewise, another study conducted in Burkina Faso attributed low case-detection to the loss of cases at each of the stages leading to diagnosis [6]. Moreover, a former study from Sierra Leone pointed out that many patients sought intermittent help from a wide range of formal and informal care practitioners [12]. Indeed, seeking care from non TB control health care practitioners led to health system delays [12], [17]. The ascertainment may explain erratic care pathways, poor use of health services as well as the challenge to case-finding. Therefore, it is crucial to instigate new strategies for early and rapid detection involving a patient-centred approach [18], incorporating a better communication pattern with the informal health system [12], and looking for a better organization and social relationship of care [19].

Although the levels of direct costs during intensive or continuation treatment are not the most important, they remain nonzero even though the TB program provides free DOTS regimen. Expenses drawn to traditional healers or private providers were comparatively modest. Whereas, expenses related to travel and to food supplements tended to be worrisome during treatment. Inappropriate care pathways including extra laboratory tests and X-rays, consultations fees, drugs and hospitalisations contribute to the risk of catastrophic TB expenditures and constitute effective financial barriers for the poor. Among our rural population, the financial pressure is catastrophically high and, for instance, far above the 10% threshold at which a household is considered to be at risk of catastrophic health expenditure [10], [20], [21].

While Africa is the only region not on track to reach the Millennium Development Goals for TB (STOP TB partnership, E-alert 17/07/2012), our study represents a real potential to feed proposals to the Stop TB Partnership initiative and prepare the next issue of the TB care and control program. To enhance success of TB strategies, countries must move towards affordable access to prevention, diagnostic and treatment services. They must focus their efforts to alleviate poverty and promote social protection interventions [22], [23]. There is still a crucial need to better understand patient barriers to care in developing countries [4], [22], [23]. Very few studies have evaluated the costs of TB care among patients in Africa [4] and so far, economic impact of TB at the household level remains an understudied area in West African countries. Furthermore, there is a lack of field data focusing on vulnerable populations such as patients living in rural areas [4]. Our study helped to fill this triple gap using a comprehensive approach and highlighted key areas generating financial barriers.

### Study limitations

The study has a number of limitations. First, convenient sampling was used focusing on a rural area which does not include any of the main cities of Burkina Faso. It is thus not fully representative of the entire country. However this focus on a poor and rural area does not appear to be a limitation as this population is particularly vulnerable to TB and directly affected by potential financial barriers. Second, bias may have been caused by the parallel development of other diseases distinct from TB but affecting the treatment. In the case of the co-infection HIV/TB, we compared overall direct costs among TB patients respectively living with HIV+ or HIV− whose medians corresponded to US$93.8 and US$89.3. Statistical tests did not show a significant difference. Third, despite the close support provided by the FORESA field officers, the careful preparation of the questionnaire (incl. cross-checking questions) and the sound training of the interviewers, some sensitive data —such as the full collection of the expenses in kind, the actual payments to traditional healers, and the possible bribes requested by health workers— may have been underestimated. However, the field knowledge of the FORESA project was an asset for the conduction of such a complex survey. Finally, relevant issues such as indirect or intangible costs were not reported in this paper. For instance, the extent of indirect costs −whose median achieved 45 workdays lost by both the patient and his guardian, corresponding to a loss of US$22.6 if we refer to the national poverty line [10]−, confirms the need to consider them. To provide comprehensive view of the economic burden of TB, those issues need to be further investigated.

## Conclusions

This study reports on the substantial and prohibitive direct cost-burden associated with TB in rural Burkina Faso. It shows that although TB diagnosis and treatment services are free, patients face erratic patient pathways (i.e. delays, non-relevant care-seeking behaviours) that uselessly worsen the direct economic burden of TB. This implies that policy-makers should find new ways to improve operationalization of international health policy. As obstacles to TB diagnosis and treatment may include economic, socio-cultural as well as organisational care supply dimensions, comprehensive interventions must be implemented involving the various care providers (incl. traditional healers), caregivers, and guardians around the patients. Tackling erratic patient pathways through improved patient-centred management should offer a promising area of progress. Whether in terms of redefining the free TB package or rationalizing the care pathway, serious efforts must be undertaken to make “free” health care more affordable for the patients. Locally relevant for TB, this case study in Burkina Faso has a real potential to document how program weaknesses can be solved. All TB programs —and more, since other international health policies are concerned— should adopt this type of approach to move towards universal health coverage.
